# COVID-19 and cognitive function: Evidence for increased processing speed variability in COVID-19 survivors and multifaceted impairment with long-COVID symptoms

**DOI:** 10.1192/j.eurpsy.2023.25

**Published:** 2023-05-12

**Authors:** Krupa Vakani, Martina Ratto, Anna Sandford-James, Elena Antonova, Veena Kumari

**Affiliations:** 1 Division of Psychology, Department of Life Sciences, College of Health, Medicine and Life Sciences, Brunel University London, Uxbridge, UK; 2 Centre for Cognitive and Clinical Neuroscience, College of Health, Medicine and Life Sciences, Brunel University London, Uxbridge, UK; 3 Being Well Group, Sheffield, UK; 4 The Scale Up Collective, London, UK

**Keywords:** Cognitive function, COVID-19, long-COVID, mental health, well-being

## Abstract

**Background:**

There is increasing evidence for cognitive function to be negatively impacted by COVID-19. There is, however, limited research evaluating cognitive function pre- and post-COVID-19 using objective measures.

**Methods:**

We examined processing speed, attention, working memory, executive function and memory in adults (≤69 years) with a history of COVID-19 (*n* = 129, none acutely unwell), compared to those with no known history of COVID-19 (*n* = 93). We also examined cognitive changes in a sub-group of COVID (*n* = 30) and non-COVID (*n* = 33) participants, compared to their pre-COVID-19 pandemic level.

**Results:**

Cross-sectionally, the COVID group showed significantly larger intra-individual variability in processing speed, compared to the non-COVID group. The COVID sub-group also showed significantly larger intra-individual variability in processing speed, compared to their pre-COVID level; no significant change occurred in non-COVID participants over the same time scale. Other cognitive indices were not significantly impacted in the cross-sectional or within-subjects investigations, but participants (*n* = 20) who had needed hospitalisation due to COVID-19 showed poor attention and executive function relative to those who had not required hospitalisation (*n* = 109). Poor health and long-COVID symptoms correlated with poor cognitive function across domains in the COVID group.

**Conclusions:**

The findings indicate a limited cognitive impact of COVID-19 with only intra-individual variability in processing speed being significantly impacted in an adult UK sample. However, those who required hospitalisation due to COVID-19 severity and/or experience long-COVID symptoms display multifaceted cognitive impairment and may benefit from repeated cognitive assessments and remediation efforts.

## Introduction

A growing body of evidence indicates widespread brain and cognitive changes in people with a history of coronavirus disease 2019 (COVID-19), including those who did not show severe symptoms and did not require hospitalisation [1, 2]. According to a systematic review [3], approximately 15–40% of COVID-19 survivors, compared to people without a history of COVID-19, show abnormal performance in one or more cognitive domain(s). More recent cross-sectional studies also indicate attention concentration [4, 5], processing speed [5], memory [6], visuospatial processing [4], executive function [4–6] and general cognitive ability [2] to be negatively impacted by COVID-19. Crivelli et al. [7] in their review of 27 studies observed impaired attention, executive functions and memory in adults who had been assessed at some point, ranging from the acute phase to 7 months after the COVID-19 infection. Most of the existing studies with an objective assessment of cognitive function, however, have utilised cross-sectional designs and focused on adults in late adulthood (mean age across 27 studies = 56.05 years, [7]) who may be particularly vulnerable to negative impacts of COVID-19 [8]. Furthermore, poor cognitive function itself has been linked to greater COVID-19 infection severity and mortality [9], raising the possibility that some of the COVID-19-related cognitive effects may be explained by pre-COVID-19 differences between COVID-19 and non-COVID groups.

The only study published to date (*n* = 785, age range: 51–81 years, Biobank cohort data, United Kingdom (UK) [10]) to use objective measures of cognitive function both pre- and post-COVID-19 reported a slight impairment in processing speed and executive function (as assessed by the Trail Making Test Trails A and B completion time, respectively) at 141 days, on average, from the COVID-19 diagnosis. There was no significant impact of COVID-19 history on eight other cognitive indices derived from six cognitive tests. Furthermore, many COVID-19 survivors report anxiety, depression, sleep difficulties and post-traumatic stress disorder (PTSD) [3, 11, 12] which could cause or exacerbate cognitive difficulties reported by COVID-19 survivors. For many people, COVID-19 also has lasting effects, commonly referred to as long-COVID [13]. In the UK, an estimated 1.9 million people have self-reported long-COVID symptoms at 4 weeks post-infection [14]. A large study (*N* = 236,379) reported neuropsychiatric diagnosis in 33.62% of patients 6-months post-infection, and this prevalence rate rose to 46.2% for patients who had received intensive care [15]. Although some of these consequences may be due to pre-existing medical and/or psychiatric conditions [16], it seems likely that COVID-19 itself results in short- and long-term neuropsychological symptoms for some people [17], and cognitive disruption may be more salient in association with long-COVID symptoms.

The main aims of the present study, therefore, were to examine: (i) the effects of COVID-19 history on cognitive function in the UK residents of working age (18–69 years); and (ii) the associations of long-COVID symptoms as well as physical and psychological well-being with cognitive function post COVID-19 diagnosis. To achieve these aims, we conducted a cross-sectional investigation of cognitive function and health in individuals with a confirmed COVID-19 diagnosis compared to those with no COVID-19 history (COVID and non-COVID groups, respectively) followed by a longitudinal investigation of participants in the COVID and non-COVID groups for whom pre-COVID-19 pandemic cognitive function data were available through an existing database. Based on the findings of previous reviews [3, 7], we expected multifaceted cognitive impairment, with the same cognitive indices being impacted by COVID-19 history in both the cross-sectional and longitudinal investigations. We further expected reduced physical and mental well-being in the COVID compared to the non-COVID group, and explored whether cognitive profiles associated with COVID-19 are explained, at least in part, by poor health and well-being. Lastly, we expected long-COVID symptoms to be associated with reduced cognitive function and poor well-being.

## Methods

### Participants and design

The cross-sectional investigation involved 222 adults (mean age = 38.70, *SD* = 12.08, range: 18–69): 129 with a COVID-19 diagnosis (COVID group) and 93 with no known/confirmed COVID-19 diagnosis (non-COVID group) (see Supplementary Table S1 for the demographic characteristics). The longitudinal investigation involved 63 of these 222 adults, who had pre-COVID-19 pandemic cognitive function data available via MyCognition [18]. Participants were recruited via social media platforms and MyCognition. Recruitment via MyCognition was conducted in two stages. First, a large group within the MyCognition database who had been assessed since 2017 (*N* = 2894) were invited to participate if they self-reported a confirmed COVID-19 diagnosis. An invitation to participate was then extended to adults with pre-COVID-19 cognitive data who self-reported no COVID-19 history. Participant testing period was March 2021–February 2022 for the COVID group and March 2021–March 2022 for the non-COVID group (recruitment of non-COVID participants stopped after the pandemic-related restrictions in the UK were fully lifted).

The study was approved by the University Research Ethics Committee (26518-A-Sep/2021–34167-1). All participants provided written consent and received £10 (Amazon voucher) for their time.

### Measures and procedure

Demographic, physical and psychological well-being data were collected using self-report measures administered via Qualtrics (an online survey tool), taking ~45 min in total. The demographic items included age, sex, ethnicity, education, socio-economic status, existing mental and physical illnesses, and medication use. In addition, COVID participants were asked about their COVID-19 diagnosis, acute symptoms, subjective cognitive impairment (via a single question “Do you believe your cognitive functioning has been impacted due to your diagnosis of COVID-19?”) and chronic long-COVID symptoms at the time of participation. Cognitive data were collected via the MyCognition PRO mobile application, taking ~15 min.

#### Physical and psychological well-being

Physical and psychological well-being were assessed using three self-rated scales:

*Short Form Health Survey-36* (SF-36) [19]: SF-36 is a 36-item scale measuring physical, social and emotional functioning, and quality of life through eight dimensions: physical functioning, physical health, emotional problems, energy, emotional well-being, social functioning, pain and general health.

*The Depression, Anxiety and Stress Scale-21* (DASS-21) [20]: DASS-21 is a 21-item scale assessing levels of depression (dysphoria, hopelessness, devaluation of life, self-deprecation, lack of interest, anhedonia, inertia), anxiety (autonomic arousal, skeletal muscle effects, situational anxiety, anxious affect) and stress (levels of chronic non-specific arousal such as problems with relaxation and emotional overactions).

*Pittsburgh Sleep Quality Index* (PSQI) [21]: PSQI is a 19-item, four-point Likert scale assessing daytime dysfunction, use of sleeping medication, sleep disturbances, habitual sleep efficiency, sleep duration, sleep latency and subjective sleep score.

#### Cognitive function

Cognitive function was assessed online using a self-administered online assessment tool (MyCognition [MyCQ], https://www.mycognition.com/). The MyCQ tool comprises of digital versions of commonly utilised neuropsychological tests validated against the Cambridge Neuropsychological Automated Test Battery [22, 23] and assesses processing speed, attention, working memory, executive function and memory domains [24].

*Processing speed* was assessed using a Simple Reaction Time (RT) task, requiring participants to tap the circle button as quickly as possible when a red circle is presented on the screen (presentation time = 1 s, inter-stimulus interval = 3 s, 30 stimuli in total). Response accuracy (RA; % correct), average RT (ms) and intra-individual variability in RT were examined.

*Attention* was assessed using a Choice Reaction Time task, requiring participants to tap either the circle or triangle button depending on what shape is presented on the screen. There are 30 trials in total, and each stimulus (circle or triangle) is presented for 1 s, with a 3 s inter-stimulus interval. RA (% correct) and average RT (ms) for correct answers were examined.

*Working Memory* was assessed using the 2-Back task. Participants are asked to tap “Yes” or “No” depending on whether the picture presented to them on the screen (household objects, food and drink items) matches the picture shown two screens back (50 trials in total). RA (% correct) was used to index task performance.

*Executive Function* was assessed using the Trail-Making B task, requiring tapping a number and a letter in an ascending and alphabetical order, respectively, to produce an alternating sequence (e.g., 1, A, 2, B). The task has 25 trials (13 numbers, 12 letters). RA (% correct moves) and total task completion time (ms) were examined.

*Memory* was assessed using a Visual Recognition Memory task. Participants are presented with a set of 24 pictures (each picture for 2 s, inter-stimulus interval = 1 s) and instructed to remember them. They are then presented with 96 pictures, including 24 pictures presented earlier, and asked to tap either “Yes” or “No” depending on whether they remember seeing the picture earlier. RA (% correct) was used to index task performance.

### Statistical analysis

For the cross-sectional investigation, we first compared the COVID and non-COVID groups on age and body mass index (BMI) (separately) using a 2 (Group: COVID, non-COVID) × 2 (Sex: Males, Females) analysis of variance (ANOVA). Group differences in each of the health and cognitive variables were examined using a 2 (Group) × 2 (Sex) ANOVA, followed by 2 (Group) × 2 (Sex) analyses of co-variance (ANCOVA), covarying for age, given that the COVID group, on average, was found to be older than the non-COVID group (Table 1). For the two cognitive variables showing a significant Group effect (see Results), further (exploratory) ANOVAs were run with Ethnicity (White British vs. all other ethnicities) included as an additional between-subjects factor. Any significant interactions were followed up with post hoc comparisons using paired or independent sample *t*-tests as appropriate. Effect sizes, where reported, are partial eta squared (*η_p_*^2^; the proportion of variance associated with a factor). In the COVID group, the relationships of cognitive variables with the overall long COVID-19 symptom load (a sum total of individual symptom ratings) were examined using Pearson’s correlations, and with each of the long-COVID symptoms (rated 0–7) explored using Spearman rank order correlations. Pearson’s correlations were also used to explore the relationships between all cognitive variables and the physical and mental health measures in the entire sample, and in the COVID and non-COVID groups separately.

**Table 1.** Descriptive statistics and group differences (ANOVA and ANCOVA results) in the demographic, mental health and well-being measures for the cross-sectional investigation

	COVID group Mean (SD)				Non-COVID group Mean (SD)			
	Male (*n* = 23)	Female (*n* = 106)	Total (*N* = 129)	Range	Male (*n* = 32)	Female (*n* = 61)	Total (*N* = 93)	Range
Demographics								
Age	42.43	40.49	40.84	19–64	34.81	36.21	35.73	18–69
	(10.99)	(11.25)	(11.19)		(12.31)	(12.95)	(12.68)	
BMI^a^	27.71	29.43	29.12	15.24–86.57	27.32	26.05	26.50	14.53–56.81
	(5.80)	(10.52)	(9.85)		(7.07)	(5.39)	(6.02)	
Physical health status (SF-36)								
Physical functioning	64.35	46.30	49.51	0–100	95.30	86.46	89.50	20–100
	(29.75)	(32.51)	(32.67)		(10.99)	(20.70)	(18.38)	
Physical health	28.26	24.76	25.39	0–100	78.13	75.82	76.61	0–100
	(40.81)	(39.42)	(39.53)		(35.21)	(39.78)	(38.09)	
Emotional problems	49.28	37.11	39.28	0–100	73.96	54.10	60.93	0–100
	(48.06)	(43.23)	(44.18)		(39.47)	(46.41)	(44.94)	
Energy/fatigue	33.48	19.04	21.61	0–80	52.86	42.92	46.34	0–95
	(19.47)	(19.86)	(20.87)		(21.80)	(22.77)	(22.81)	
Emotional well-being	62.39	51.47	53.42	4–96	66.88	61.05	63.05	4–100
	(17.88)	(21.98)	(21.65)		(18.96)	(21.85)	(20.98)	
Social functioning	55.98	49.06	50.29	0–100	73.05	71.11	71.77	0–100
	(27.66)	(25.74)	(26.12)		(25.02)	(26.47)	(25.86)	
Pain	67.39	51.49	54.32	0–100	87.81	76.23	80.22	0–100
	(29.47)	(28.18)	(28.95)		(18.78)	(25.50)	(23.95)	
General health	57.17	44.67	46.90	0–95	69.22	60.57	63.55	20–100
	(22.66)	(21.42)	(22.08)		(21.03)	(20.82)	(21.18)	
Mental health (DASS-21)								
Depression	11.57	15.08	14.45	0–42	8.69	11.48	10.52	0–42
	(9.14)	(10.71)	(10.50)		(9.79)	(11.41)	(10.91)	
Anxiety	9.04	10.77	10.47	0–38	5.62	7.93	7.14	0–36
	(7.28)	(8.83)	(8.57)		(7.28)	(8.27)	(7.98)	
Stress	12.87	14.87	14.51	0–40	8.75	15.41	13.12	0–42
					(7.46)	(10.82)	(10.26)	
	(8.50)	(9.67)	(9.47)					
Sleep quality (PSQI)								
Sleep quality	1.57	1.76	1.73	0–3	1.19	1.44	1.35	0–3
	(0.79)	(0.72)	(0.74)		(0.59)	(0.83)	(0.76)	
Sleep latency	2.09	2.06	2.06	0–3	1.38	1.59	1.52	0–3
	(1.04)	(0.95)	(0.97)		(1.04)	(1.10)	(1.08)	
Sleep duration^b^	1.35	1.03	1.09	0–3	0.84	0.90	0.88	0–3
	(0.98)	(0.88)	(0.91)		(0.77)	(0.93)	(0.87)	
Sleep efficiency^c^	1.43	1.43	1.43	0–3	0.72	0.76	0.75	0–3
	(1.17)	(1.13)	(1.14)		(1.05)	(1.01)	(1.02)	
Sleep disturbance	1.48	1.66	1.63	0–3	1.20	1.41	1.33	0–3
	(0.67)	(0.62)	(0.63)		(0.54)	(0.56)	(0.56)	
Sleep medication^d^	0.26	0.75	0.66	0–3	0.44	0.20	0.28	0–3
	(0.75)	(1.24)	(1.18)		(0.98)	(0.70)	(0.81)	
Daytime dysfunction	1.17	1.45	1.40	0–3	0.84	1.11	1.02	0–3
	(0.78)	(0.86)	(0.85)		(0.68)	(0.80)	(0.77)	
Global score	9.22	10.06	9.91	2–18	6.41	7.31	7.00	1–20
	(3.70)	(3.61)	(3.63)		(3.61)	(3.89)	(3.80)	

	ANOVA *F*(1,218) (*p*) *η_p_*^2^			ANCOVA (covarying for age) *F*(1,217) (*p*) *η_p_*^2^			
	Group effect	Sex	Group × sex	Age effect	Group effect	Sex	Group × sex
Demographics							
Age	10.00 **(0.002)**	0.02 (0.885)	0.790 (0.375)	n/a	n/a	n/a	n/a
	*0.44*	*0.0*	*0.004*				
BMI^a^	1.96 (0.163)	0.03 (0.868)	1.22 (0.271)	10.94 **(0.001)**	0.44 (0.506)	0.05 (0.816)	1.69 (0.195)
	*0.01*	*0.0*	*0.01*	*0.05*	*0.002*	*0.0*	*0.01*
Physical health status (SF-36)							
Physical functioning	68.56 **(<0.001)**	9.81 **(0.002)**	1.15 (0.284)	3.56 (0.068)	60.17 **(<0.001)**	10.02 **(0.002)**	1.41 (0.237)
	*0.24*	*0.04*	*0.01*	*0.02*	*0.22*	*0.04*	*0.01*
Physical health	66.28 **(<0.001)**	0.22 (0.640)	0.01 (0.924)	1.48 (0.226)	59.52 **(<0.001)**	0.23 (0.631)	0.03 (0.866)
	*0.23*	*0.001*	*0.0*	*0.01*	*0.22*	*0.001*	*0.0*
Emotional problems	8.87 **(0.003)**	5.24 **(0.023)**	0.30 (0.583)	3.67 (0.057)	11.09 **(0.001)**	5.22 **(0.023)**	0.45 (0.506)
	*0.04*	*0.02*	*0.001*	*0.02*	*0.05*	*0.02*	*0.002*
Energy/fatigue	41.59 **(<0.001)**	13.20 **(<0.001)**	0.45 (0.503)	0.04 (0.852)	40.08 **(<0.001)**	13.13 **(<0.001)**	0.43 (0.512)
	*0.16*	*0.06*	*0.002*	*0.0*	*0.16*	*0.06*	*0.002*
Emotional well-being	4.40 **(0.037)**	6.23 **(0.013)**	0.58 (0.448)	8.66 **(0.004)**	7.30 **(0.007)**	6.31 **(0.013)**	0.35 (0.553)
	*0.02*	*0.03*	*0.003*	*0.04*	*0.03*	*0.03*	*0.002*
Social functioning	22.43 **(<0.001)**	1.15 (0.284)	0.36 (0.547)	1.97 (0.162)	24.36 **(<0.001)**	1.13 (0.290)	0.27 (0.604)
	*0.09*	*0.01*	*0.002*	*0.01*	*0.1*	*0.01*	*0.001*
Pain	29.01 **(<0.001)**	10.75 **(0.001)**	0.27 (0.607)	2.24 (0.136)	24.68 **(<0.001)**	10.90 **(0.001)**	0.37 (0.545)
	*0.12*	*0.05*	*0.001*	*0.01*	*0.10*	*0.05*	*0.002*
General health	17.08 **(<0.001)**	9.78 **(0.002)**	0.33 (0.569)	1.78 (0.183)	14.20 **(<0.001)**	9.90 **(0.002)**	0.42 (0.516)
	*0.07*	*0.04*	*0.001*	*0.01*	*0.06*	*0.04*	*0.002*
Mental health (DASS-21)							
Depression	3.69 (0.056)	3.49 (0.063)	0.05 (0.831)	4.24 **(0.041)**	5.40 **(0.021)**	3.46 (0.064)	0.01 (0.927)
	*0.02*	*0.02*	*0.0*	*0.02*	*0.02*	*0.02*	*0.0*
Anxiety	5.62 **(0.019)**	2.34 (0.127)	0.05 (0.826)	11.19 **(0.001)**	9.44 **(0.002)**	2.35 (0.127)	0.18 (0.671)
	*0.03*	*0.01*	*0.0*	*0.05*	*0.04*	*0.01*	*0.001*
Stress	1.38 (0.242)	8.07 **(0.005)**	2.34 (0.128)	14.71 **(<0.001)**	3.95 **(0.048)**	8.36 **(0.004)**	3.26 (0.073)
	*0.01*	*0.04*	*0.01*	*0.06*	*0.02*	*0.04*	*0.02*
Sleep quality (PSQI)							
Sleep quality	8.78 **(0.003)**	3.70 (0.056)	0.06 (0.812)	0.65 (0.422)	7.44 **(0.007)**	3.73 (0.055)	0.04 (0.850)
	*0.04*	*0.02*	*0.0*	*0.003*	*0.03*	*0.02*	*0.0*
Sleep latency	13.34 **(<0.001)**	0.33 (0.567)	0.58 (0.448)	0.82 (0.367)	14.13 **(<0.001)**	0.32 (0.574)	0.66 (0.417)
	*0.06*	*0.002*	*0.003*	*0.004*	*0.06*	*0.001*	*0.003*
Sleep duration^b^	5.00 **(0.026)**	0.86 (0.355)	1.79 (0.183)	0.91 (0.341)	3.95 **(0.048)**	0.84 (0.361)	1.62 (0.204)
	*0.02*	*0.004*	*0.01*	*0.004*	*0.02*	*0.004*	*0.01*
Sleep efficiency^c^	15.02 **(<0.001)**	0.01 (0.905)	0.02 (0.898)	12.54**(<0.001)**	10.32 **(0.002)**	0.02 (0.899)	0.01 (0.939)
	*0.07*	*0.0*	*0.0*	*0.06*	*0.05*	*0.0*	*0.0*
Sleep disturbance	8.24 **(0.005)**	4.60 **(0.033)**	0.05 (0.831)	0.02 (0.904)	7.70 **(0.006)**	4.58 **(0.033)**	0.04 (0.838)
	*0.04*	*0.02*	*0.0*	*0.0*	*0.03*	*0.02*	*0.0*
Sleep medication^d^	1.31 (0.253)	0.57 (0.451)	4.94 **(0.027)**	2.45 (0.119)	0.64 (0.426)	0.60 (0.439)	5.38 **(0.021)**
	*0.01*	*0.003*	*0.02*	*0.01*	*0.003*	*0.003*	*0.02*
Daytime dysfunction	6.72 **(0.010)**	4.55 **(0.034)**	0.0 (0.976)	3.77 (0.054)	8.75 **(0.003)**	4.53 **(0.034)**	0.01 (0.932)
	*0.03*	*0.02*	*0.0*	*0.02*	*0.04*	*0.02*	*0.0*
Global score	22.41 **(<0.001)**	2.21 (0.139)	0.003 (0.955)	1.38 (0.241)	19.25 **(<0.001)**	2.25 (0.135)	0.0 (0.989)
	*0.09*	*0.01*	*0.0*	*0.01*	*0.08*	*0.01*	*0.0*

SF-36 (Short Form Health Survey-36): The response ranges between two- and six-point ordered Likert scales. Raw scores are transformed to produce a score between 0 and 100 for each dimension. The higher the score the better the overall health (19.47). Internal reliability in this sample: overall scale, Cronbach’s *a* = 0.96; all subscales, Cronbach’s *a* > 0.8, except *a* = 0.74 for Social Functioning.

DASS-21 (The Depression, Anxiety and Stress Scale-21): Each item is rated by participants on a four-point scale according to how often in the past week it applied to them, ranging from “Did not apply to me at all” (0) to “Applied to me very much or most of the time” (3). Higher scores indicate higher levels (severity) of symptoms. Internal reliability in this sample: Depression, Cronbach’s *a =* 0.93; Anxiety, Cronbach’s *a* = 0.82; Stress, Cronbach’s *a =*0.88.

PSQI (Pittsburgh Sleep Quality Index): Participants answer the PSQI questions by relating them to their past month (21). Each component is scored between “No difficulty” (0) to “Severe difficulty” (3) and tallied up to yield a total score (range 0–21). Higher scores indicate poor sleep quality. Internal reliability in this sample: Global score, Cronbach’s *a* = 0.76.

a Sample size reduced by 2 (non-COVID).

b Sample size reduced by 1 (COVID).

c Sample size reduced by 7 (5 COVID, 2 non-COVID).

d Sample size reduced by 2 (COVID).

For the longitudinal (within-subjects) investigation, the COVID and non-COVID groups were compared on age and BMI using independent sample *t*-tests (sex not analysed due to relatively small number of males). The effect of COVID-19 diagnosis on each of the cognitive variables was then examined using a 2 (Group: COVID, non-COVID) × 2 (Time: Pre-COVID, Post-COVID) ANOVA with Group as a between-subjects and Time as a within-subject factor. Given that poor cognitive function has been linked to more severe acute COVID-19 and hospitalisation [9], the relationship between the overall long-COVID symptom load and pre-pandemic cognitive data in the longitudinal COVID sub-sample (*n* = 29) was also examined using Pearson’s correlations.

All analyses were performed using the Statistical Package for Social Sciences (for Windows, version 28; IBM, New York, NY). The data distribution on all variables met the assumptions of parametric statistical procedures. Alpha level for testing the significance of effects was maintained at *p* ≤ 0.05.

## Results

### Cross-sectional investigation

#### Sample characteristics

The majority of participants in both the COVID (*n* = 129) and non-COVID (*n* = 93) groups were White British, held a Bachelor’s degree or above and were in some form of employment. The COVID group was, on average, significantly older (Table 1), and had more people with at least one physical health problem (*n* = 58; 44.96%; most commonly related to lungs), compared to the non-COVID group (*n* = 21; 22.58%). Of various mental health conditions, anxiety, depression and insomnia were most commonly reported by both groups (Supplementary Table S1).

Within the COVID group, 20 participants (15.5%) had been hospitalised (Supplementary Tables S2 and S3). The most prevalent acute symptom (recalled retrospectively) was a high temperature (76.7%). At study entry (mean number of days since diagnosis = 263, *SD* = 192.16, range:20–714), a large proportion of the sample reported subjective cognitive impairment (78.3%), reduced psychological well-being (77.5%), and one or more long-COVID symptoms, most commonly exhaustion/fatigue (88.4%). The overall long-COVID symptom load, however, was not significantly correlated with the number of days since diagnosis [*r*(125) = 0.057, *p* = 0.527] or age [*r*(126) = 0.092, *p* = 0.299]. Nineteen of 20 hospitalised participants (95%) reported subjective cognitive impairment, and 18 (90%) reported reduced psychological well-being.

#### Mental health and psychological well-being in COVID versus non-COVID participants

There were significant main effects of Group in ANOVA analyses (Table 1), with the COVID group having significantly poorer health (SF-36), higher anxiety (DASS-21), and lower sleep quality (PSQI), compared to the non-COVID group. The ANCOVA analyses, with age as a covariate, retained these effects and, in addition, indicated significantly higher depression and stress levels (DASS-21) in the COVID, compared to the non-COVID group (Table 1).

There were significant sex differences in physical functioning, emotional problems, energy, emotional well-being, pain and general health (SF-36), stress (DASS-21), as well as sleep disturbance and daytime dysfunction (PSQI), indicating poorer health and psychological well-being in females compared to males. There were, however, no significant Group × Sex interactions (Table 1), except for sleep medication [in females, greater use of sleep medications by COVID compared to non-COVID group, *t*(163) = 3.65, *p* < 0.001].

Lastly, age was a significant covariate (in ANCOVAs) for BMI, emotional well-being (SF-36), depression, anxiety and stress (DASS-21), as well as sleep efficiency and daytime dysfunction (PSQI), indicating poorer health and psychological well-being with older age.

#### Cognitive function in COVID versus non-COVID participants

There were significant main effects of Group indicating significantly greater intra-individual variability in processing speed (*p* = 0.015) and lower attention RA (*p* = 0.022) in the COVID compared to non-COVID group (Table 2). The Group effect remained significant for processing speed variability (*p* = 0.034) but lost formal significance for attention RA (*p* = 0.052) when covarying for age, with ANCOVAs additionally revealing longer RTs being associated with older age (Table 2). Ethnicity did not show any main or interactive effects (Table 2).

**Table 2.** Descriptive statistics and group differences (ANOVA and ANCOVA results) in cognitive measures for the cross-sectional investigation

		COVID group Mean (SD)				Non-COVID group Mean (SD)			
		Male (*n* = 23)	Female (*n* = 106)	Total (*N* = 129)	Range	Male (*n* = 32)	Female (*n* = 61)	Total (*N* = 93)	Range
Processing speed^a^	Response accuracy (%)	94.87	95.87	95.68	57.14–100	96.36	96.66	96.56	56.67–100
		(9.02)	(6.56)	(7.07)		(8.08)	(5.15)	(6.26)	
	RT (correct responses, ms)	352.04	382.45	376.57	275–686	351.32	355.47	354.05	253–746
		(77.90)	(81.66)	(81.52)		(66.93)	(76.96)	(73.35)	
	RT variability (SD of RT)	84.52	87.21	86.69	22–205	64.00	76.18	72.03	25–182
		(40.85)	(42.21)	(41.80)		(34.74)	(39.11)	(37.93)	
Attention^b^	Response accuracy (%)	94.55	95.23	95.11	51.28–100	97.06	98.16	97.78	73.33–100
		(10.43)	(8.18)	(8.58)		(5.52)	(4.12)	(4.66)	
	RT (correct responses, ms)	476.76	514.16	507.39	344–830	484.03	461.19	469.15	321–898
		(101.50)	(93.76)	(95.84)		(114.70)	(87.16)	(97.60)	
Working memory^c^	Response accuracy (%)	92.73	91.85	92.00	52–100	95.07	93.69	94.18	60–100
		(5.57)	(8.89)	(8.40)		(4.62)	(7.44)	(6.59)	
Executive function^d^	Accuracy (%)	95.40	94.64	94.78	58.14–100	93.80	95.51	94.94	58.14–100
		(5.59)	(7.55)	(7.22)		(9.71)	(7.96)	(8.57)	
	Completion time (ms)	29,217.00	34,046.42	33,185.36	11,775–162,669	31,398.80	29,385.68	30,056.72	12050–97180
		(11,514.15)	(20,399.68)	(19,172.91)		(11,740.80)	(12,823.20)	(12,443.24)	
Memory^e^	Recognition accuracy (%)	90.26	89.25	89.42	54.17–98.96	91.15	91.59	91.44	58.33–100
		(6.96)	(8.88)	(8.56)		(7.99)	(8.40)	(8.22)	

		ANOVA*F*(1, 218) (*p*) *η_p_*^2^			ANCOVA (covarying for age)*F*(1, 217) (*p*) *η_p_*			
		Group effect	Sex	Group × sex	Age effect	Group effect	Sex	Group × sex
Processing speed^a^	Response accuracy (%)	1.11 (0.294)	0.35 (0.553)	0.11 (0.746)	0.41 (0.522)	0.78 (0.377)	0.35 (0.556)	0.09 (0.770)
		*0.01*	*0.002*	*0.001*	*0.002*	*0.004*	*0.002*	*0.0*
	RT (correct responses, ms)	1.23 (0.269)	1.91 (0.168)	1.11 (0.295)	5.59 **(0.019)**	0.34 (0.563)	2.00 (0.159)	1.37 (0.243)
		*0.01*	*0.01*	*0.01*	*0.03*	*0.002*	*0.01*	*0.01*
	RT variability (SD of RT)	6.00 **(0.015)**	1.33 (0.250)	0.54 (0.462)	1.35 (0.247)	4.57 **(0.034)**	1.35 (0.246)	0.47 (0.495)
		*0.03*	*0.01*	*0.003*	*0.01*	*0.02*	*0.01*	*0.002*
Attention^b^	Response accuracy (%)	5.33 **(0.022)**	0.57 (0.451)	0.03 (0.856)	2.59 (0.109)	3.83 (0.052)	0.59 (0.443)	0.05 (0.830)
		*0.03*	*0.003*	*0.0*	*0.01*	*0.02*	*0.003*	*0.0*
	RT (correct responses, ms)	2.10 (0.149)	0.21 (0.645)	3.64 (0.058)	31.01**(<0.001)**	0.18 (0.670)	0.21 (0.649)	4.68 **(0.032)**
		*0.01*	*0.001*	*0.02*	*0.13*	*0.001*	*0.001*	*0.02*
Working memory^c^	Response accuracy (%)	2.84 (0.093)	0.82 (0.366)	0.04 (0.842)	0.01 (0.940)	2.74 (0.099)	0.81 (0.368)	0.04 (0.839)
		*0.01*	*0.004*	*0.0*	*0.0*	*0.01*	*0.004*	*0.0*
Executive function^d^	Accuracy (%)	0.08 (0.774)	0.14 (0.705)	0.97 (0.326)	2.93 (0.088)	0.02 (0.896)	0.14 (0.712)	0.73 (0.394)
		*0.0*	*0.001*	*0.004*	*0.01*	*0.0*	*0.001*	*0.003*
	Completion time (ms)	0.21 (0.645)	0.28 (0.601)	1.62 (0.204)	0.40 (0.527)	0.09 (0.768)	0.27 (0.604)	1.73 (0.189)
		*0.001*	*0.001*	*0.01*	*0.002*	*0.0*	*0.001*	*0.01*
Memory^e^	Recognition accuracy (%)	1.43 (0.233)	0.05 (0.832)	0.29 (0.592)	0.34 (0.559)	1.07 (0.302)	0.05 (0.825)	0.33 (0.567)
		*0.01*	*0.0*	*0.001*	*0.002*	*0.01*	*0.0*	*0.002*

*Note*: Further (exploratory) analyses of processing speed RT variability and attention response accuracy (%) with Ethnicity (White British vs. All Other Ethnicities) included as an additional between-subjects factor retained the main effects of Group (processing speed RT variability, *p* = 0.006; attention RA, *p* = 0.042) but yielded no significant main or interactive effects involving Ethnicity (all *p* values > 0.341).

a Sample size reduced by 12 (10 COVID, 2 non-COVID).

b Sample size reduced by 17 (13 COVID, 4 non-COVID).

c Sample size reduced by 5 (2 COVID, 3 non-COVID).

d Sample size reduced by 3 (non-COVID).

e Sample size reduced by 2 (COVID).

Participants who had been hospitalised had longer attention RTs (*p* = 0.005) and lower executive function RA (%) (*p* = 0.012) than those who did not require hospitalisation (Table 3). These effects remained significant when covarying for age, despite older age being associated with poorer performance on both measures.

**Table 3.** Descriptive statistics and group differences between COVID hospitalised versus non-hospitalised sample (ANOVA and ANCOVA results) in cognitive measures for the cross-sectional investigation

		Hospitalised COVID participants *n* = 20		Non-hospitalised COVID participants *n* = 109		ANOVA *F*(1,127) (*p*) *η_p_*^2^	ANCOVA (covarying for age) *F*(1,126) (*p*) *η_p_*^2^	
		Mean (SD)	Range	Mean (SD)	Range	Hospital effect	Age effect	Hospital effect
Processing speed^a^	Response accuracy (%)	94.09 (9.42)	71.43–100	95.98 (6.55)	57.14–100	1.13 (0.289) *0.01*	0.16 (0.693) *0.001*	1.20 (0.275) *0.01*
	RT (correct responses, ms)	393.00 (87.94)	301–611	373.45 (80.33)	275–686	0.92 (0.340) *0.01*	0.45 (0.506) *0.004*	0.77 (0.381) *0.01*
	RT variability (SD of RT)	93.95 (32.79)	52–186	85.31 (43.30)	22–205	0.68 (0.411) *0.01*	0.17 (0.684) *0.001*	0.60 (0.441) *0.01*
Attention^b^	Response accuracy (%)	94.93 (7.13)	78.79–100	95.14 (8.87)	51.28–100	0.01 (0.921) *0.0*	0.27 (0.608) *0.002*	0.002 (0.967) *0.0*
	RT (correct responses, ms)	562.84 (70.79)	438–714	496.53 (96.63)	344–830	8.07 **(0.005)** *0.07*	11.89 **(0.001)** *0.1*	6.62 **(0.011)** *0.06*
Working memory^c^	Response accuracy (%)	90.31 (11.38)	52–100	92.39 (7.75)	52–100	0.97 (0.327) *0.01*	0.42 (0.518) *0.003*	1.12 (0.293) *0.01*
Executive function	Accuracy (%)	91.06 (11.64)	58.14–100	95.46 (5.91)	71.43–100	6.52 **(0.012)** *0.05*	8.06 **(0.005)** *0.06*	8.84 **(0.004)** *0.07*
	Completion time (ms)	41,747.45 (30,405.35)	14,575–162,669	31,614.34 (16,030.19)	11,775–13,1328	4.86 **(0.029)** *0.04*	0.03 (0.875) *0.0*	4.83 **(0.030)** *0.04*
Memory^c^	Recognition accuracy (%)	88.35 (10.46)	54.17–98.96	89.62 (8.20)	57.14–98.96	0.37 (0.543) *0.003*	0.13 (0.718) *0.001*	0.42 (0.518) *0.003*

a Sample size reduced by 10 (1 hospitalised, 9 non-hospitalised).

b Sample size reduced by 13 (1 hospitalised, 12 non-hospitalised).

c Sample size reduced by 2 (non-hospitalised).

#### Association between cognitive functions and long-COVID symptoms

Executive function task completion time, the RTs during processing speed and attention tasks, and attention RA variables were most commonly correlated, with small-to-medium effect sizes, with individual long-COVID symptoms, especially arrythmia, chest pain and headaches (Supplementary Table S4). The overall long-COVID symptom load was significantly associated with poor performance on all tasks, with small-to-medium-sized correlations (Table 4).

**Table 4.** Associations (Pearson correlation coefficients) between the cognitive variables and the total long COVID-19 symptom load in the COVID participants

	Processing speed			Attention		Working memory	Executive function		Memory
	Response accuracy (%) *r (p) n* [CI]	RT correct responses (ms) *r (p) n* [CI]	RT variability(SD of RT) *r (p) n* [CI]	Response accuracy (%) *r (p) n* [CI]	RT correct responses (ms) *r (p) n* [CI]	Accuracy (%) *r (p) n* [CI]	Response accuracy (%) *r (p) n* [CI]	Completion time (ms) *r (p) n* [CI]	Recognition accuracy (%) *r (p) n* [CI]
Cross-sectional investigation									
Long COVID symptom load	−0.246 **(0.007)** 118	0.315 **(0.001)** 118	0.209 **(0.023)** 118	−0.273 **(0.003)** 115	0.368 **(<0.001)** 115	−0.220 **(0.013)** 126	−0.240 **(0.006)** 128	0.289 **(0.001)** 128	−0.253 **(0.004)** 126
	[−0.409, −0.068]	[0.142, 0.469]	[0.029, 0.376]	[−0.434, −0.095]	[0.198, 0.516]	[−0.380, −0.047]	[−0.397, −0.069]	[0.122, 0.440]	[−0.410, −0.082]
Longitudinal (within-subjects) investigation – cognitive function at study entry									
Long COVID symptom load	Cognitive function at study entry								
	−0.158 (0.422) 28	−0.062 (0.753) 28	0.043 (0.828) 28	−0.207 (0.290) 28	0.129 (0.513) 28	−0.175 (0.363) 29	−0.012 (0.950) 29	0.373 **(0.046)** 29	−0.157 (0.417) 29
	[−0.502, 0.229]	[−0.425, 0.318]	[−0.336, 0.410]	[−0.539, 0.180]	[−0.256, 0.479]	[−0.509, 0.205]	[−0.377, 0.356]	[0.008, 0.651]	[−0.495, 0.222]
	Pre-pandemic cognitive function								
	0.109 (0.573) 29	0.089 (0.644) 29	0.021 (0.912) 29	−0.075 (0.704) 28	0.079 (0.690) 28	0.104 (0.598) 28	−0.365 (0.052) 29	0.502 **(0.005)** 29	−0.378 **(0.043)** 29
	[−0.268, 0.457]	[−0.287, 0.441]	[−0.348, 0.385]	[−0.436, 0.307]	[−0.303, 0.439]	[−0.280, 0.459]	[−0.645, 0.002]	[0.166, 0.734]	[−0.654, −0.013]

#### Association between cognitive function, mental health and well-being

SF-36 dimensions correlated with many cognitive variables, especially executive function, with poorer health being associated with poorer performance (Table 5). Depression, anxiety and stress correlated negatively with executive function. Sleep disturbance was associated with poor performance in processing speed, attention and executive function. These associations were generally stronger in the COVID, relative to non-COVID, group (Table 5).

**Table 5.** Associations (Pearson correlation coefficients) of the cognitive variables with health and well-being measures for the cross-sectional investigation

	Processing speed				Attention				Working memory				Executive function				Memory	
	Response accuracy (%) *r* (*p*)		RT correct responses (ms) *r* (*p*)		RT variability (SD of RT)*r* (*p*)		Response accuracy (%) *r* (*p*)		RT correct responses (ms) *r* (*p*)		Accuracy (%) *r* (*p*)		Response accuracy (%) *r* (*p*)		Completion time (ms) *r* (*p*)		Recognition accuracy (%) *r* (*p*)	
	COV + *n* = 119	COV−*n* = 91	COV + *n* = 119	COV−*n* = 91	COV + *n* = 119	COV−*n* = 91	COV + *n* = 116	COV−*n* = 89	COV + *n* = 116	COV−*n* = 89	COV + *n* = 127	COV−*n* = 90	COV + *n* = 129	COV−*n* = 90	COV + *n* = 129	COV−*n* = 90	COV + *n* = 127	COV−*n* = 93
Physical health status (SF-36)																		
Physical functioning	0.109 (0.238)	0.134 (0.205)	−0.317 **(<0.001)**	−0.123 (0.244)	−0.159 (0.085)	−0.224 **(0.033)**	0.132 (0.158)	−0.005 (0.961)	−0.368 **(<0.001)**	−0.065 (0.546)	0.230 **(0.009)**	0.137 (0.199)	0.248 (**0.005**)	0.066 (0.536)	−0.287 **(0.001)**	−0.042 (0.697)	0.213 **(0.016)**	0.003 (0.979)
Physical health	0.106 (0.253)	0.140 (0.186)	−0.190 **(0.039)**	0.022 (0.835)	−0.096 (0.297)	−0.026 (0.807)	0.046 (0.625)	−0.042 (0.695)	−0.304 **(0.001)**	−0.032 (0.766)	0.135 (0.131)	0.041 (0.704)	0.180 **(0.041)**	0.079 (0.459)	−0.122 (0.167)	−0.001 (0.993)	0.065 (0.465)	−0.027 (0.794)
Emotional problems	0.151 (0.101)	−0.018 (0.865)	−0.004 (0.963)	0.235 **(0.025)**	−0.058 (0.528)	0.159 (0.133)	0.009 (0.926)	−0.118 (0.271)	−0.100 (0.283)	0.219 **(0.040)**	0.021 (0.814)	0.093 (0.381)	0.242 **(0.006)**	−0.067 (0.531)	−0.079 (0.376)	0.197 (0.062)	0.112 (0.210)	−0.169 (0.106)
Energy/fatigue	0.093 (0.312)	0.160 (0.129)	−0.243 **(0.008)**	0.024 (0.823)	−0.119 (0.196)	−0.023 (0.827)	0.125 (0.182)	−0.033 (0.762)	−0.215 **(0.020)**	−0.058 (0.588)	0.065 (0.470)	0.101 (0.344)	0.126 (0.156)	0.071 (0.508)	−0.108 (0.225)	0.034 (0.752)	−0.021 (0.817)	0.023 (0.827)
Emotional well-being	0.095 (0.306)	0.183 (0.083)	−0.001 (0.988)	0.118 (0.265)	−0.049 (0.595)	−0.084 (0.426)	0.018 (0.848)	−0.038 (0.724)	−0.038 (0.685)	0.102 (0.343)	0.051 (0.570)	0.119 (0.263)	0.185 **(0.036)**	0.204 (0.053)	0.013 (0.883)	−0.062 (0.559)	−0.029 (0.747)	0.208 **(0.045)**
Social Functioning	0.150 (0.103)	0.131 (0.218)	−0.134 (0.145)	0.174 (0.099)	−0.066 (0.473)	−0.085 (0.423)	0.007 (0.942)	0.174 (0.103)	−0.169 (0.070)	0.125 (0.245)	0.234 **(0.008)**	0.157 (0.139)	0.241 **(0.006)**	0.169 (0.111)	−0.126 (0.155)	−0.077 (0.473)	0.076 (0.393)	0.011 (0.916)
Pain	0.174 (0.059)	0.163 (0.123)	−0.341 **(<0.001)**	−0.068 (0.523)	−0.239 **(0.009)**	−0.270 **(0.010)**	0.115 (0.219)	−0.037 (0.727)	−0.293 **(0.001)**	−0.041 (0.703)	0.205 **(0.021)**	0.083 (0.435)	0.182 **(0.039)**	0.148 (0.165)	−0.145 (0.101)	−0.145 (0.173)	0.168 (0.059)	−0.054 (0.609)
General health	0.069 (0.454)	0.149 (0.160)	−0.312 **(0.001)**	−0.115 (0.279)	−0.161 (0.081)	−0.160 (0.129)	0.116 (0.214)	0.027 (0.802)	−0.322 **(<0.001)**	−0.151 (0.157)	0.004 (0.965)	0.127 (0.234)	0.112 (0.205)	0.160 (0.131)	−0.086 (0.335)	−0.114 (0.285)	0.169 (0.058)	−0.022 (0.832)
Mental health (DASS-21)																		
Depression	−0.080 (0.384)	−0.088 (0.407)	0.003 (0.978)	−0.117 (0.269)	0.025 (0.786)	0.017 (0.872)	−0.017 (0.854)	0.017 (0.878)	0.030 (0.747)	−0.107 (0.319)	−0.181 **(0.042)**	−0.003 (0.977)	−0.172 **(0.052)**	−0.252 **(0.017)**	−0.025 (0.777)	0.068 (0.526)	0.042 (0.636)	−0.072 (0.494)
Anxiety	−0.178 **(0.053)**	−0.187 (0.076)	0.065 (0.485)	−0.116 (0.274)	0.121 (0.188)	−0.002 (0.834)	−0.113 (0.226)	−0.006 (0.959)	0.016 (0.861)	−0.076 (0.481)	−0.135 (0.129)	−0.091 (0.394)	−0.398 **(<0.001)**	−0.165 (0.119)	0.160 (0.071)	−0.033 (0.757)	−0.152 (0.088)	−0.027 (0.801)
Stress	−0.065 (0.482)	−0.129 (0.223)	−0.005 (0.959)	−0.116 (0.271)	0.008 (0.933)	0.108 (0.307)	−0.088 (0.350)	−0.048 (0.655)	−0.013 (0.888)	−0.114 (0.289)	−0.173 **(0.052)**	−0.023 (0.826)	−0.221 **(0.012)**	−0.264 **(0.012)**	0.012 (0.896)	0.138 (0.195)	0.009 (0.917)	−0.072 (0.490)
Sleep quality (PSQI)																		
Sleep quality	−0.172 (0.062)	−0.024 (0.823)	0.098 (0.289)	−0.087 (0.412)	0.167 (0.069)	0.019 (0.854)	−0.102 (0.274)	0.145 (0.175)	0.072 (0.440)	−0.037 (0.729)	−0.081 (0.363)	−0.034 (0.747)	−0.059 (0.506)	0.009 (0.930)	−0.078 (0.377)	0.019 (0.856)	0.039 (0.663)	0.192 (0.065)
Sleep latency	−0.080 (0.390)	0.033 (0.757)	0.133 (0.149)	−0.071 (0.504)	0.121 (0.190)	−0.049 (0.641)	−0.109 (0.242)	0.193 (0.069)	0.049 (0.601)	−0.147 (0.169)	−0.089 (0.319)	0.009 (0.931)	−0.051 (0.566)	−0.174 (0.100)	0.010 (0.908)	0.076 (0.477)	−0.064 (0.472)	0.141 (0.178)
Sleep duration	−0.145 (0.118)^a^	−0.114 (0.284)	0.001 (0.990)^a^	−0.091 (0.389)	0.069 (0.458)^a^	0.118 (0.264)	−0.050 (0.598)^a^	−0.059 (0.584)	0.089 (0.343)^a^	0.014 (0.900)	−0.155 (0.083)^a^	0.035 (0.744)	−0.135 (0.128)^a^	−0.040 (0.705)	0.025 (0.776)^a^	0.001 (0.989)	−0.189 **(0.035)** ^a^	0.011 (0.915)
Sleep efficiency	−0.108 (0.251)^d^	−0.018 (0.870)^c^	0.157 (0.095)^d^	−0.181 (0.092)^c^	0.132 (0.162)^d^	−0.020 (0.856)^c^	−0.064 (0.505)^d^	0.103 (0.346)^c^	0.212 **(0.025)** ^d^	−0.117 (0.285)^c^	−0.047 (0.611)^d^	0.181 (0.094)^c^	0.048 (0.596)^d^	−0.076 (0.486)^c^	−0.033 (0.713)^d^	0.017 (0.874)^c^	−0.142 (0.118)^d^	0.149 (0.162)^c^
Sleep disturbance	−0.188 **(0.041)**	−0.117 (0.268)	0.262 **(0.004)**	0.123 (0.246)	0.143 (0.120)	0.156 (0.139)	−0.161 (0.083)	0.158 (0.140)	0.236 **(0.011)**	0.081 (0.449)	−0.110 (0.218)	−0.028 (0.794)	−0.190 **(0.031)**	−0.152 (0.152)	0.198 **(0.024)**	0.147 (0.165)	−0.150 (0.092)	0.099 (0.347)
Sleep medication	0.094 (0.316)^c^	0.056 (0.596)	0.146 (0.116)^c^	−0.034 (0.746)	−0.063 (0.498)^c^	−0.044 (0.680)	−0.084 (0.376)^c^	0.113 (0.291)	0.164 (0.082)^c^	−0.005 (0.965)	0.079 (0.383)^c^	0.119 (0.265)	−0.011 (0.906)^c^	−0.093 (0.383)	0.053 (0.553)^c^	0.002 (0.985)	−0.163 (0.069)^c^	−0.038 (0.718)
Daytime dysfunction	−0.110 (0.232)	−0.052 (0.623)	0.021 (0.817)	−0.046 (0.662)	0.004 (0.963)	−0.043 (0.687)	−0.010 (0.917)	0.088 (0.411)	0.079 (0.396)	−0.035 (0.747)	0.005 (0.958)	−0.022 (0.836)	−0.174 **(0.048)**	−0.046 (0.670)	0.042 (0.633)	−0.067 (0.530)	−0.061 (0.493)	0.135 (0.197)
Global PSQI score	−0.154 (0.094)	−0.028 (0.794)	0.195 **(0.034)**	−0.083 (0.435)	0.126 (0.173)	0.027 (0.801)	−0.118 (0.209)	0.159 (0.137)	0.212 **(0.022)**	−0.052 (0.631)	−0.090 (0.312)	0.050 (0.638)	−0.128 (0.148)	−0.105 (0.326)	0.050 (0.573)	0.040 (0.707)	−0.194 **(0.029)**	0.137 (0.192)

	Processing speed			Attention		Working memory	Executive function		Memory
	Response accuracy (%) *r* (*p*)	RT correct responses (ms) *r* (*p*)	RT variability (SD of RT) *r* (*p*)	Response accuracy (%) *r* (*p*)	RT correct responses (ms) *r* (*p*)	Accuracy (%) *r* (*p*)	Response accuracy (%) *r* (*p*)	Completion time (ms) *r* (*p*)	Recognition accuracy (%) *r* (*p*)
	All *N* = 210	All *N* = 210	All *N* = 210	All *N* = 205	All *N* = 205	All *N* = 217	All *N* = 219	All *N*= 219	All *N* = 220
Physical health status (SF-36)									
Physical functioning	0.130 (0.060)	−0.289 **(<0.001)**	−0.243 **(<0.001)**	0.190 **(0.006)**	−0.324 **(<0.001)**	0.247 **(<0.001)**	0.152 **(0.024)**	−0.246 **(<0.001)**	0.189 **(0.005)**
Physical health	0.135 **(0.051)**	−0.166 **(0.016)**	−0.156 **(0.024)**	0.117 (0.096)	−0.260 **(<0.001)**	0.160 **(0.018)**	0.119 (0.079)	−0.120 (0.076)	0.088 (0.194)
Emotional problems	0.095 (0.170)	0.056 (0.417)	−0.013 (0.853)	0.017 (0.807)	−0.007 (0.920)	0.076 (0.267)	0.101 (0.138)	−0.014 (0.841)	0.023 (0.732)
Energy/fatigue	0.137 **(0.047)**	−0.180 **(0.009)**	−0.153 **(0.027)**	0.150 **(0.032)**	−0.216 **(0.002)**	0.135 **(0.047)**	0.092 (0.175)	−0.096 (0.158)	0.057 (0.403)
Emotional well-being	0.140 **(0.043)**	0.016 (0.820)	−0.099 (0.152)	0.042 (0.546)	−0.022 (0.757)	0.100 (0.140)	0.190 **(0.005)**	−0.029 (0.674)	0.090 (0.183)
Social functioning	0.156 **(0.024)**	−0.065 (0.350)	−0.137 **(0.048)**	0.118 (0.093)	−0.112 (0.110)	0.240 **(<0.001)**	0.196 **(0.004)**	−0.135 **(0.046)**	0.091 (0.181)
Pain	0.181 **(0.009)**	−0.277 **(<0.001)**	−0.300 **(<0.001)**	0.146 **(0.037)**	−0.255 **(<0.001)**	0.211 **(0.002)**	0.154 **(0.022)**	−0.169 **(0.012)**	0.129 (0.057)
General health	0.117 (0.092)	−0.266 **(<0.001)**	−0.211 **(0.002)**	0.144 **(0.039)**	−0.295 **(<0.001)**	0.093 (0.173)	0.128 (0.058)	−0.119 (0.079)	0.128 (0.058)
Mental health (DASS-21)									
Depression	−0.093 (0.177)	−0.020 (0.774)	0.053 (0.4434)	−0.038 (0.590)	0.003 (0.965)	−0.136 **(0.046)**	−0.207 **(0.002)**	0.020 (0.771)	−0.026 (0.698)
Anxiety	−0.190 **(0.006)**	0.017 (0.808)	0.093 (0.182)	−0.112 (0.110)	0.015 (0.834)	−0.142 **(0.036)**	−0.291 **(<0.001)**	0.116 (0.088)	−0.121 (0.073)
Stress	−0.095 (0.170)	−0.043 (0.533)	0.060 (0.385)	−0.082 (0.240)	−0.045 (0.520)	−0.123 (0.071)	−0.241 **(<0.001)**	0.057 (0.402)	−0.033 (0.624)
Sleep quality (PSQI)									
Sleep quality	−0.123 (0.076)	0.054 (0.436)	0.143 **(0.038)**	−0.069 (0.328)	0.068 (0.334)	−0.094 (0.167)	−0.029 (0.668)	−0.023 (0.741)	0.071 (0.296)
Sleep latency	−0.048 (0.486)	0.082 (0.234)	0.094 (0.175)	−0.064 (0.360)	0.015 (0.830)	−0.082 (0.226)	−0.109 (0.107)	0.054 (0.427)	−0.006 (0.934)
Sleep duration	−0.140 **(0.043)** ^a^	−0.015 (0.827)^a^	0.112 (0.107)^a^	−0.069 (0.328)^a^	0.075 (0.286)^a^	−0.103 (0.133)^a^	−0.093 (0.170)^a^	0.028 (0.679)^a^	−0.120 (0.077)^a^
Sleep efficiency	−0.085 (0.226)^b^	0.077 (0.274)^b^	0.115 (0.101)^b^	−0.067 (0.345)^b^	0.134 (0.061)^b^	−0.020 (0.771)^b^	−0.011 (0.872)^b^	0.011 (0.868)^b^	−0.061 (0.376)^b^
Sleep disturbance	−0.171 **(0.013)**	0.233 **(0.001)**	0.183 **(0.008)**	−0.108 (0.124)	0.206 **(0.003)**	−0.113 (0.097)	−0.170 **(0.012)**	0.197 **(0.003)**	−0.081 (0.230)
Sleep medication	0.072 (0.304)^c^	0.104 (0.136)^c^	−0.029 (0.677)^c^	−0.062 (0.381)^c^	0.124 (0.077)^c^	0.065 (0.344)^c^	−0.040 (0.560)^c^	0.055 (0.417)^c^	−0.139 **(0.041)** ^c^
Daytime dysfunction	−0.099 (0.151)	0.025 (0.719)	0.024 (0.731)	−0.025 (0.719)	0.074 (0.290)	−0.036 (0.595)	−0.118 (0.082)	0.030 (0.656)	−0.012 (0.862)
Global PSQI score	−0.118 (0.089)	0.123 (0.074)	0.141 **(0.041)**	−0.094 (0.181)	0.154 **(0.028)**	−0.085 (0.211)	−0.113 (0.095)	0.075 (0.267)	−0.093 (0.169)

Abbreviations: COV+, COVID group; COV−, Non-COVID group; DASS-21, The Depression, Anxiety and Stress Scale-21; PSQI, Pittsburgh Sleep Quality Index; SF-36, Short Form Health Survey-36.

a Sample size reduced by 1.

b Sample size reduced by 7.

c Sample size reduced by 2.

d Sample size reduced by 5.

### Longitudinal (within-subjects) investigation

#### Sample characteristics

The sub-sample for whom pre-COVID cognitive data were available had similar sample characteristics as the whole sample (Supplementary Table S1).

#### Pre- versus post-COVID-19 cognitive function

In line with the cross-sectional findings, there was a significant Group × Time interaction in RT variability in processing speed (*p* = 0.043; Table 6), explained by greater intra-individual variability in the COVID group post-COVID-19 diagnosis (but not acutely unwell) compared to their pre-pandemic scores [*t*(28)=2.01, *p* = 0.05]; there was no such change in the non-COVID group [*t*(30)=0.75, *p* = 0.461]. Additionally, there were main effects of Time on attention task RTs (*p* = 0.001) (shorter RTs the second time) and working memory RA (*p* = 0.033) (slightly higher the second time), most likely explained by practice-related effects.

**Table 6.** Descriptive statistics and changes from pre-pandemic assessment (ANOVA results) in cognitive measures for the longitudinal investigation (sub-sample with pre-pandemic cognitive data)

		Time 1: Pre-COVID-19 pandemic Mean (SD)			Time 2: During COVID-19 pandemic Mean (SD)			ANOVA F(1,61) (*p*) *η_p_*^2^		
		COVID (*n* = 30)	Non-COVID (*n* = 33)	Total (*N* = 63)	COVID (*n* = 30)	Non-COVID (*n* = 33)	Total (*N* = 63)	Group effect	Time	Group × Time
Processing speed^a^	Response accuracy (%)	97.10	96.79	96.94	96.50	95.84	96.16	0.17 (0.681)	0.74 (0.393)	0.04 (0.846)
		(7.21)	(4.45)	(5.92)	(5.07)	(5.67)	(5.35)	*0.003*	*0.01*	*0.001*
	RT (correct responses, ms)	331.86	353.17	342.69	338.72	342.70	340.75	0.87 (0.354)	0.10 (0.748)	2.41 (0.126)
		(48.16)	(59.57)	(54.86)	(52.71)	(62.79)	(57.59)	*0.02*	*0.002*	*0.04*
	RT variability (SD of RT)	61.86	77.70	69.92	80.41	71.77	76.02	0.19 (0.667)	1.14 (0.291)	4.29 **(0.043)**
		(40.49)	(35.54)	(38.56)	(45.01)	(35.05)	(40.14)	*0.003*	*0.02*	*0.07*
Attention^b^	Response accuracy (%)	97.18	98.14	97.67	97.96	96.32	97.13	0.13 (0.722)	0.28 (0.599)	1.76 (0.190)
		(4.46)	(4.40)	(4.42)	(3.71)	(7.20)	(5.76)	*0.002*	*0.01*	*0.03*
	RT (correct responses, ms)	471.57	495.17	483.58	442.18	462.69	452.61	1.03 (0.314)	12.41 **(0.001)**	0.03 (0.861)
		(86.39)	(108.56)	(98.15)	(64.53)	(87.93)	(77.33)	*0.02*	*0.18*	*0.001*
Working memory^c^	Response accuracy (%)	92.81	94.35	93.58	95.66	95.80	95.73	0.361 (0.550)	4.78 **(0.033)**	0.51 (0.480)
		(9.22)	(6.28)	(7.86)	(4.47)	(5.05)	(4.73)	*0.01*	*0.08*	*0.01*
Executive function^d^	Accuracy (%)	97.00	96.03	96.50	97.20	94.78	95.95	2.09 (0.154)	0.33 (0.568)	0.62 (0.434)
		(4.64)	(6.30)	(5.53)	(4.06)	(7.57)	(6.20)	*0.03*	*0.01*	*0.01*
	Completion time (ms)	27173.43	32866.81	30111.95	26701.10	28917.69	27845.15	2.38 (0.128)	1.94 (0.168)	1.20 (0.277)
		(11,845.29)	(13,489.15)	(12,938.44)	(11,223.29)	(10,631.37)	(10,889.00)	*0.04*	*0.03*	*0.02*
Memory	Recognition accuracy (%)	90.38	90.16	90.27	90.57	91.23	90.92	0.02 (0.904)	0.37 (0.545)	0.18 (0.672)
		(7.92)	(6.83)	(7.31)	(7.54)	(10.25)	(9.00)	*0.0*	*0.01*	*0.003*

a Sample size reduced by 4 (1 COVID, 3 non-COVID).

b Sample size reduced by 6 (2 COVID, 4 non-COVID).

c Sample size reduced by 5 (1 COVID, 4 non-COVID).

d Sample size reduced by 1 (non-COVID).

#### Association between cognitive function and long-COVID symptoms

The higher overall long-COVID symptom load correlated with poorer executive function performance at study entry (post-Covid), as well as poorer executive function and memory in pre-pandemic cognitive data (see Table 4).

## Discussion

The main aims of this study were to examine the impact of COVID-19 on cognitive function in a UK adult sample (≤69 years), and explore the roles of physical and mental health and long-COVID symptoms in cognitive function in these individuals. The findings showed: (i) significantly larger intra-individual variability in processing speed but no significant impact of COVID-19 on other cognitive measures in our cross-sectional investigation, and a further confirmation of a negative impact of COVID-19 on processing speed variability (but no other cognitive variables) in our within-subjects investigation (pre-pandemic vs. post-COVID-19 diagnosis); (ii) poorer attention and executive function in the COVID-19 group participants who had needed hospitalisation due to COVID-19 relative to those who had not; and (iii) medium-sized negative associations of cognitive performance on all tasks with physical health and long-COVID symptoms (and relatively fewer associations of cognitive variables with anxiety and depression) in the COVID-19 group in the cross-sectional investigation, and (iv) medium-sized negative association between pre-pandemic cognitive function (executive function, memory) and long-COVID symptoms in the longitudinal sample. Further noteworthy findings were: poorer physical and mental health in the COVID relative to non-COVID group; generally reduced psychological well-being in females, relative to males; and longer RTs with increasing age.

Concerning the cognitive impact of COVID-19, our findings indicated only a limited negative impact of COVID-19 history on cognitive function in UK adults <70 years, with only processing speed variability being impacted (with a small effect size) out of nine cognitive function indices examined in both the cross-sectional and longitudinal investigations. This is consistent with the findings of the UK Biobank data-based study [10] which found significant effects of COVID-19 on only 2 (Trails A and B completion time) out of 10 cognitive function indices examined. We did not detect the impact of COVID-19 as “slower speed” (including Trails B completion time) but rather a “more variable speed” on a task where speed was emphasised. Given that Douaud et al. [10] did not examine/report intra-individual variability in processing speed, the findings of their and our study combined suggest that intra-individual variability in speed might be relatively more sensitive to COVID-19 in people aged ≤69 years, since our sample had a wider age range of younger adults (18–69) compared to Douaud et al. [10] study’s age range (51–81 years). Elevated intra-individual variability in RTs, reflecting momentary lapses in attention and/or task-irrelevant cognitions and a neural dysfunction involving multiple networks [25, 26], has also been shown to be a particularly sensitive measure in the context of ageing [27, 28], prediction of future cognitive outcomes [29], and various neurodegenerative diseases [30, 31]. A positive consideration here is that there may be scope for improving/reducing variability in processing speed using continuous cognitive training [32] or mindfulness-based approaches [33], given findings of a more stable performance in long-term meditators compared to meditation-naïve individuals [34], as well as other reports of improved processing speed following mindfulness practice [35, 36].

Against the backdrop of a limited general cognitive impact of COVID-19, our findings suggest reduced cognitive function across multiple domains in people who needed hospitalisation due to COVID-19. This is consistent with recent literature suggesting that brain and cognitive impairment may be more salient in people with a severe infection [37] or hospitalisation [38], highlighting the need for longitudinal cognitive monitoring and improvement efforts in such cohorts [39], for example using non-invasive brain stimulation [40, 41]. Furthermore, we found sizable associations between overall long-COVID symptom load and cognitive function across all domains, suggesting that affected individuals may also benefit from longitudinal cognitive monitoring and rehabilitation efforts. Interestingly, further to previous literature linking poorer cognitive function to greater acute COVID-19 severity and hospitalisation [9], our finding suggests that poorer cognitive function (executive function, memory) may also be a precursor of long-COVID symptoms. Taken together, our findings indicate that at least a part of COVID-19-related cognitive impairment in cross-sectional studies may reflect reduced pre-infection cognitive level (for various other reasons); and that the most robust short-to-medium impact of COVID-19 may be limited to a more variable processing speed. Follow-up assessments of our and other samples are crucially needed to fully chart the cognitive trajectory of COVID-19 and long-COVID.

Our further findings confirmed poorer mental health and well-being in COVID-19 survivors [3, 42] and suggest that this may continue for some time post-infection. In addition, sex differences (across groups) were observed with women reporting poorer mental health and well-being, which is also in line with previous literature on sex differences in affective disorders [43]. Lastly, our finding of longer RTs with increasing age is consistent with previous literature [44].

The limitations of the present study include: (i) most participants being White-British, limiting generalisability of the findings; (ii) significantly older participants, on average, in the COVID than non-COVID groups (due to the open recruitment strategy), though all COVID-related effects were sustained when covarying for age; and (iii) a reliance on self-report for COVID-19 diagnosis, which cannot rule out that at least some non-COVID group participants may have been pre-symptomatic when assessed. Future research should include more ethnically-diverse samples with consideration to the impact of socio-economic factors and, importantly, assess cognitive function at numerous times post-infection to understand the potential long-term cognitive recovery, especially in association with varying levels of long-COVID symptoms [45, 46].

## Conclusions

We observed a limited cognitive impact of COVID-19 with only intra-individual variability in processing speed being significantly affected (becoming less stable) in an adult UK sample. However, those who required COVID-19-related hospitalisation, did display multifaceted cognitive impairment. Furthermore, long-COVID symptoms were associated with reduced cognitive function (assessed post-COVID-19 diagnosis) but also with poorer executive function and memory prior to the COVID-19 pandemic, suggesting that poorer cognitive function may be a precursor of long-COVID symptoms. Further research is required to understand whether COVID-19 and long-COVID continue to impede cognitive function over a longer period of time.

## Supplementary material

For supplementary material accompanying this paper visit http://doi.org/10.1192/j.eurpsy.2023.25. click here to view supplementary material
