# Supplementary material for: COVID-19 and cognitive function: Evidence for increased processing speed variability in COVID-19 survivors and multifaceted impairment with long-COVID symptoms
Source: Eur Psychiatry. 2023 May 12;66(1):e43. doi: 10.1192/j.eurpsy.2023.25 (PMC10305372; doi:10.1192/j.eurpsy.2023.25)
Supplement: Supplementary file 1 [file S0924933823000251sup001.docx]

**Supplementary Materials**

**Supplementary Table 1. *Sample* *characteristics.***

|  | | **Cross-Sectional Sample** | | **Longitudinal Sub-sample** | |
| --- | --- | --- | --- | --- | --- |
|  |  | **COVID Group N=129**  **(23 M, 106 F)** | **Non-COVID Group N=93**  **(32 M, 61 F)** | **COVID Group N=30**  **(4 M, 26 F)** | **Non-COVID Group N=33**  **(7 M, 26 F)** |
|  |  | ***n* (% of N)** | | | |
| **Ethnicity** | White British | 107 (82.9%) | 41 (44.1%) | 24 (80.0%) | 28 (84.9%) |
|  | South Asian | 13 (10.1%) | 42 (45.2%) | 2 (6.7%) | 2 (6.1%) |
|  | Other Asian | 1 (0.8%) | 4 (4.3%) | 0 (0%) | 1 (3.0%) |
|  | Black British | 1 (0.8%) | 2 (2.2%) | 0 (0%) | 1 (3.0%) |
|  | Mixed Race | 6 (4.7%) | 3 (3.3%) | 3 (10.0%) | 0 (0%) |
|  | Other | 1 (0.8%) | 1 (1.1%) | 1 (3.3%) | 1 (3.0%) |
| **Educational Background** | High School | 7 (5.4%) | 5 (5.4%) | 2 (6.7%) | 4 (12.1%) |
|  | College/6th Form | 20 (15.5%) | 11 (11.8%) | 5 (16.7%) | 3 (9.1%) |
|  | Vocational Qualification | 16 (12.4%) | 7 (7.5%) | 4 (13.3.%) | 6 (18.2%) |
|  | Bachelor's Degree | 50 (38.8%) | 33 (35.5%) | 12 (40%) | 11 (33.3%) |
|  | Master's Degree | 26 (20.2%) | 31 (33.3%) | 6 (20%) | 7 (21.2%) |
|  | PhD or Higher | 7 (5.4%) | 6 (6.5%) | 0 (0%) | 2 (6.1%) |
|  | Prefer not to say | 3 (2.3%) | 0 (0%) | 1 (3.3%) | 0 (0%) |
| **Employment Status** | Employed Full-time | 61 (47.3%) | 54 (58.1%) | 15 (50.0%) | 13 (39.4%) |
|  | Employed Part-time | 26 (20.2%) | 12 (12.9%) | 5 (16.7%) | 5 (15.2%) |
|  | Student Full-time | 9 (7.0%) | 10 (10.8%) | 3 (10.0%) | 3 (9.1%) |
|  | Student Part-time | 1 (0.8%) | 2 (2.2%) | 1 (3.3%) | 1 (3.0%) |
|  | Unemployed | 1 (0.8%) | 1 (1.1%) | 1 (3.3%) | 1 (3.0%) |
|  | Retired | 2 (1.6%) | 2 (2.2%) | 1 (3.3%) | 2 (6.1%) |
|  | Semi-retired | 3 (2.3%) | 3 (3.2%) | 1 (3.3%) | 1 (3.0%) |
|  | Homemaker | 2 (1.6%) | 1 (1.1%) | 0 (0%) | 1 (3.0%) |
|  | Unable to Work | 12 (9.3%) | 2 (2.2%) | 0 (0%) | 2 (6.1%) |
|  | Other | 8 (6.2%) | 5 (5.4%) | 0 (0%) | 3 (9.1%) |
|  | Prefer not to say | 4 (3.1%) | 1 (1.1%) | 3 (10.0%) | 1 (3.0%) |
| **Physical Health Conditions** | Cancer | 5 (3.9%) | 0 (0%) | 3 (10.0%) | 0 (0%) |
|  | Diabetes | 10 (7.8%) | 5 (5.4%) | 2 (6.7%) | 1 (3.0%) |
|  | Heart Condition | 8 (6.2%) | 2 (2.2%) | 2 (6.7%) | 2 (6.1%) |
|  | Immunosuppressed | 8 (6.2%) | 2 (2.2%) | 1 (3.3%) | 1 (3.0%) |
|  | Kidney Disease | 1 (0.8%) | 0 (0%) | 0 (0%) | 0 (0%) |
|  | Liver Disease | 2 (1.6%) | 1 (1.1%) | 2 (6.7%) | 1 (3.0%) |
|  | Lung Condition | 28 (21.7%) | 7 (7.5%) | 4 (13.3%) | 3 (9.1%) |
|  | Neurological Condition | 7 (5.4%) | 1 (1.1%) | 4 (13.3%) | 1 (3.0%) |
|  | Obesity | 18 (14.0%) | 7 (7.5%) | 2 (6.7%) | 2 (6.1%) |
|  | Organ Transplantation | 1 (0.8%) | 0 (0%) | 0 (0%) | 0 (0%) |
| **Mental Health Conditions** | Anorexia Nervosa | 2 (1.6%) | 2 (2.2%) | 0 (0%) | 2 (6.1%) |
|  | Anxiety | 56 (43.4%) | 39 (41.9%) | 15 (50.0%) | 23 (69.7%) |
|  | ADHD | 3 (2.3%) | 3 (3.2%) | 2 (6.7%) | 1 (3.0%) |
|  | Depression | 48 (37.2%) | 27 (29%) | 12 (40.0%) | 18 (54.5%) |
|  | Eating Disorder(s) | 8 (6.2%) | 4 (4.3%) | 2 (6.7%) | 3 (9.1%) |
|  | Insomnia | 29 (22.5%) | 13 (14%) | 4 (13.3%) | 9 (27.3%) |
|  | OCD | 7 (5.4%) | 5 (5.4%) | 2 (6.7%) | 3 (9.1%) |
|  | Panic Disorder | 10 (7.8%) | 7 (7.5%) | 3 (10.0%) | 7 (21.2%) |
|  | Personality Disorder | 4 (3.1%) | 1 (1.1%) | 1 (3.3%) | 1 (3.0%) |
|  | Phobias | 9 (7.0%) | 7 (7.5%) | 3 (10.0%) | 4 (12.1%) |
|  | PTSD | 18 (14.0%) | 6 (6.5%) | 5 (16.7%) | 6 (18.2%) |
|  | Psychosis | 2 (1.6%) | 3 (3.2%) | 0 (0%) | 3 (9.1%) |
|  | Other | 2 (1.6%) | 1 (1.1%) | 0 (0%) | 0 (0%) |

M = males, F = females.

**Supplementary Table 2. *COVID-19 diagnosis history and symptoms in COVID group participants.***

|  | | **Cross-Sectional Investigation** | | **Longitudinal**  **Investigation** | |
| --- | --- | --- | --- | --- | --- |
|  |  | ***n*** | **% of total** | ***n*** | **% of sub-sample total** |
| **Confirmed COVID-19 Diagnosis** | | 129 | - | 30 | - |
| **COVID-19 Diagnosis Date** | November '19 | 1 | 0.8% | 1 | 3.3% |
|  | January '20 | 2 | 1.6% | 2 | 6.7% |
|  | March '20 | 13 | 10.1% | 1 | 3.3% |
|  | April '20 | 11 | 8.5% | 2 | 6.6% |
|  | May '20 | 2 | 1.6% | 0 | 0% |
|  | June '20 | 1 | 0.8% | 1 | 3.3% |
|  | August ‘20 | 1 | 0.8% | 0 | 0% |
|  | September '20 | 5 | 3.9% | 1 | 3.3% |
|  | October '20 | 9 | 7.0% | 2 | 6.6% |
|  | November '20 | 16 | 12.4% | 5 | 16.5% |
|  | December '20 | 23 | 17.8% | 8 | 26.6% |
|  | January '21 | 15 | 11.6% | 3 | 9.9% |
|  | February '21 | 1 | 0.8% | 0 | 0% |
|  | March '21 | 2 | 1.6% | 0 | 0% |
|  | May ‘21 | 2 | 1.6% | 0 | 0% |
|  | June '21 | 2 | 1.6% | 1 | 3.3% |
|  | July '21 | 2 | 1.6% | 0 | 0% |
|  | August ‘21 | 2 | 1.6% | 0 | 0% |
|  | September '21 | 4 | 3.1% | 2 | 6.6% |
|  | October '21 | 5 | 3.9% | 1 | 3.3% |
|  | November ‘21 | 3 | 2.3% | 0 | 0% |
|  | December ‘21 | 3 | 2.3% | 0 | 0% |
|  | January '22 | 3 | 2.3% | 0 | 0% |
| **Hospitalisation due to COVID** | | 20 | 15.5% | 2 | 6.7% |
| **Acute COVID-19 Symptoms** | Temperature | 99 | 76.7% | 18 | 60% |
|  | Dry Cough | 86 | 66.6% | 17 | 56.6% |
|  | Loss of Taste and/or Smell | 83 | 64.3% | 21 | 70% |
|  | Other | 83 | 64.3% | 16 | 69.6% |
| **Subjective Cognitive Function Impairment** | | 101 | 78.3% | 12 | 41.4% |
| **Subjective Reduced Psychological Well-being** | | 100 | 77.5% | 13 | 43.3% |
| **Chronic COVID-19 Symptoms**  **(Long COVID)** | Abdominal pain | 49 | 38.0% | 3 | 10.3% |
|  | Arrhythmia | 72 | 55.8% | 5 | 17.2% |
|  | Body chills | 61 | 47.3% | 3 | 10.3% |
|  | Breathing problems | 91 | 70.5% | 11 | 37.9% |
|  | Chest pain | 69 | 53.5% | 4 | 13.7% |
|  | Chilblains | 19 | 14.7% | 1 | 1.6% |
|  | Confusion/delirium | 77 | 59.7% | 8 | 27.5% |
|  | Diarrhoea | 44 | 34.1% | 4 | 13.7% |
|  | Dry cough | 46 | 35.7% | 8 | 27.5% |
|  | Exhaustion/fatigue | 114 | 88.4% | 19 | 65.4% |
|  | Hallucinations | 19 | 14.7% | 0 | 0% |
|  | Headaches | 95 | 73.6% | 15 | 51.7% |
|  | Insomnia | 101 | 78.3% | 17 | 58.5% |
|  | Irritability | 94 | 72.9% | 13 | 44.7% |
|  | Lack of appetite | 62 | 48.1% | 7 | 24.0% |
|  | Loss of taste and/or smell | 47 | 36.4% | 7 | 24.0% |
|  | Mild cognitive problems | 107 | 82.9% | 16 | 55.0% |
|  | Muscle/body ache | 97 | 75.2% | 16 | 55.0% |
|  | Sore eyes/conjunctivitis | 59 | 45.7% | 7 | 24.0% |
|  | Sore throat | 47 | 36.4% | 5 | 17.2% |
|  | Temperature | 36 | 27.9% | 4 | 13.7% |
|  | Vomiting/nausea | 34 | 26.4% | 4 | 13.7% |
|  | Other | 29 | 22.5% | 5 | 17.2% |

**Supplementary Table 3. *Descriptive statistics and group differences between COVID hospitalised versus non-hospitalised sample (ANOVA and ANCOVA results) in the demographic, mental health and well-being measures for the cross-sectional investigation.***

|  | | **Hospitalised COVID Participants *n* = 20** | | **Non-hospitalised COVID Participants *n* = 109** | | **ANOVA F(1, 127) (*p*) *η_p_²*** | **ANCOVA (covarying for age) F(1, 126) (*p*) *η_p_²*** | |
| --- | --- | --- | --- | --- | --- | --- | --- | --- |
|  |  | **Mean (SD)** | **Range** | **Mean (SD)** | **Range** | **Hospital Effect** | **Age Effect** | **Hospital Effect** |
| **Demographics** | Age | 44.25 (11.36) | 23-64 | 40.21 (11.10) | 19-64 | 2.22 (0.138) *0.02* | n/a | n/a |
|  | BMI | 32.21 (10.98) | 18.59-62.67 | 28.56 (9.57) | 15.24-86.57 | 2.34 (0.128) *0.02* | 6.67 **(0.011)** *0.05* | 1.47 (0.228) *0.01* |
| **Physical Health Status (SF-36)** | Physical functioning | 36.00 (18.82) | 10-85 | 51.99 (34.10) | 0-100 | 4.15 **(0.044)** *0.03* | 1.09 (0.299) *0.01* | 3.55 (0.062) *0.03* |
|  | Physical health | 10.00 (23.51) | 0-100 | 28.21 (41.26) | 0-100 | 3.66 (0.058) *0.03* | 1.40 (0.239) *0.01* | 3.05 (0.083) *0.02* |
|  | Emotional problems | 8.33 (23.88) | 0-100 | 44.95 (44.86) | 0-100 | 12.67 **(0.001)** *0.09* | 9.18 **(0.003)** *0.07* | 16.30 **(<0.001)** *0.12* |
|  | Energy/fatigue | 15.75 (15.75) | 0-55 | 22.69 (21.56) | 0-80 | 1.88 (0.172) *0.02* | 0.03 (0.865) *0.0* | 1.78 (0.185) *0.01* |
|  | Emotional well-being | 49.80 (18.01) | 4-72 | 54.08 (22.26) | 8-96 | 0.66 (0.418) *0.01* | 11.64 **(0.001)** *0.09* | 1.65 (0.201) *0.01* |
|  | Social functioning | 36.88 (20.06) | 0-75 | 52.75 (26.43) | 0-100 | 6.51 **(0.012)** *0.05* | 3.14 (0.079) *0.02* | 7.75 **(0.006)** *0.06* |
|  | Pain | 40.75 (26.43) | 0-90 | 56.81 (28.81) | 0-100 | 5.38 **(0.022)** *0.04* | 0.36 (0.549) *0.003* | 4.91 **(0.029)** *0.04* |
|  | General health | 42.50 (19.90) | 15-75 | 47.71 (22.45) | 0-95 | 0.94 (0.334) *0.01* | 0.06 (0.805) *0.0* | 0.86 (0.357) *0.01* |
| **Mental Health (DASS-21)** | Depression | 15.30 (10.51) | 2-42 | 14.29 (10.54) | 0-42 | 0.15 (0.695) *0.001* | 3.34 (0.070) *0.03* | 0.40 (0.528) *0.003* |
|  | Anxiety | 11.90 (7.77) | 0-26 | 10.20 (8.72) | 0-38 | 0.66 (0.418) *0.01* | 19.75 **(<0.001)** *0.14* | 2.09 (0.151) *0.02* |
|  | Stress | 18.10 (8.81) | 6-36 | 13.85 (9.47) | 0-40 | 3.47 (0.065) *0.03* | 18.85 **(<0.001)** *0.13* | 6.45 **(0.012)** *0.05* |
| **Sleep Quality (PSQI)** | Sleep quality | 1.80 (0.70) | 0-3 | 1.72 (0.75) | 0-3 | 0.22 (0.640) *0.002* | 0.31 (0.581) *0.002* | 0.29 (0.592) *0.002* |
|  | Sleep latency | 2.25 (0.91) | 0-3 | 2.03 (0.98) | 0-3 | 0.90 (0.346) *0.01* | 0.57 (0.451) *0.01* | 1.07 (0.302) *0.01* |
|  | Sleep duration^a^ | 1.42 (1.12) | 0-3 | 1.03 (0.86) | 0-3 | 3.11 (0.080) *0.02* | 0.003 (0.959) *0.0* | 3.06 (0.083) *0.02* |
|  | Sleep efficiency^b^ | 1.94 (1.16) | 0-3 | 1.34 (1.11) | 0-3 | 4.50 **(0.036)** *0.04* | 6.59 **(0.012)** *0.05* | 3.56 (0.061) *0.03* |
|  | Sleep disturbance | 1.75 (0.55) | 1-3 | 1.61 (0.64) | 0-3 | 0.90 (0.345) *0.01* | 0.04 (0.843) *0.0* | 0.98 (0.337) *0.01* |
|  | Sleep medication^c^ | 0.42 (1.02) | 0-3 | 0.70 (1.21) | 0-3 | 0.92 (0.339) *0.01* | 0.04 (0.841) *0.0* | 0.95 (0.331) *0.01* |
|  | Daytime dysfunction | 1.80 (0.89) | 1-3 | 1.33 (0.83) | 0-3 | 5.30 **(0.023)** *0.04* | 4.35 **(0.039)** *0.03* | 6.69 **(0.011)** *0.05* |
|  | Global score | 11.10 (3.92) | 2-16 | 9.69 (3.55) | 2-18 | 2.59 (0.110) *0.02* | 0.01 (0.914) *0.0* | 2.57 (0.111) *0.02* |

^a^ Sample size reduced by 1 (hospitalised); ^b^ Sample size reduced by 5 (2 hospitalised, 3 non-hospitalised); ^c^ Sample size reduced by 2 (1 hospitalised, 1 non-hospitalised).

SF-36: Short Form Health Survey-36; DASS-21: The Depression, Anxiety and Stress Scale-21; PSQI: Pittsburgh Sleep Quality Index.

**Supplementary Table 4. *Correlation between the cognitive variables and the individual chronic long COVID-19 symptoms in the COVID participants.***

| **Cross-Sectional Investigation** | | | | | | | | | |
| --- | --- | --- | --- | --- | --- | --- | --- | --- | --- |
| ***Individual Long-COVID Symptoms*** | **Processing Speed**  (*n=119)* | | | **Attention**  (*n*=116) | | **Working Memory** (*n*=127) | **Executive Function**  (*n*=129) | | **Memory**  (*n*=127) |
|  | Response accuracy (%)  *rho (p)* | RT correct responses (ms)  *rho (p)* | RT variability  (SD of RT) *rho (p)* | Response accuracy (%) *rho (p)* | RT Correct Responses (ms) *rho (p)* | Accuracy (%) *rho (p)* | Response accuracy (%) *rho (p)* | Completion time (ms) *rho (p)* | Recognition accuracy (%) *rho (p)* |
| **Abdominal pain** | -0.075 (0.420) | 0.177 (0.055) | 0.089 (0.336) | -0.174 (0.062) | 0.119 (0.204) | -0.076 (0.395) | -0.139 (0.115) | 0.158 (0.074) | -0.090 (0.314) |
| **Arrhythmia** | -0.208 **(0.023)** | 0.208 **(0.023)** | 0.195 **(0.033)** | -0.255 **(0.006)** | 0.253 **(0.006)** | -0.166 (0.063) | -0.270 **(0.002)** | 0.293 **(0.001)** | -0.199 **(0.025)** |
| **Body chills** | -0.053 (0.564) | 0.227 **(0.013)** | 0.125 (0.174) | -0.155 (0.097) | 0.216 **(0.020)** | -0.172 (0.053) | -0.086 (0.332) | 0.193 **(0.029)** | 0.015 (0.871) |
| **Breathing problems** | -0.099 (0.285) | 0.202 **(0.027)** | 0.167 (0.070) | -0.146 (0.118) | 0.237 **(0.011)** | -0.130 (0.144) | -0.234 **(0.008)** | 0.278 **(0.001)** | -0.169 (0.058) |
| **Chest pain** | -0.091 (0.323) | 0.301 **(0.001)** | 0.171 (0.063) | -0.237 **(0.010)** | 0.344 **(<0.001)** | -0.225 **(0.011)** | -0.293 **(0.001)** | 0.344 **(<0.001)** | -0.193 **(0.030)** |
| **Chilblains** | -0.136 (0.142) | 0.016 (0.862) | 0.105 (0.258) | -0.059 (0.531) | 0.070 (0.456) | -0.256 (0.528) | 0.009 (0.918) | 0.125 (0.159) | -0.159 (0.075) |
| **Confusion/delirium** | -0.147 (0.111) | 0.160 (0.082) | 0.197 **(0.032)** | -0.226 **(0.015)** | 0.256 **(0.006)** | -0.119 (0.181) | -0.126 (0.153) | 0.173 **(0.050)** | -0.036 (0.685) |
| **Diarrhoea** | -0.087 (0.347) | 0.265 **(0.004)** | 0.121 (0.191) | -0.081 (0.390) | 0.125 (0.181) | -0.215 **(0.015)** | -0.089 (0.314) | 0.263 **(0.003)** | -0.106 (0.235) |
| **Dry cough** | -0.006 (0.951) | 0.066 (0.473) | 0.106 (0.251) | -0.107 (0.252) | 0.057 (0.546) | -0.119 (0.184) | -0.077 (0.387) | 0.220 **(0.012)** | -0.081 (0.367) |
| **Exhaustion/fatigue** | -0.073 (0.430) | 0.280 **(0.002)** | 0.155 (0.091) | -0.165 (0.076) | 0.283 **(0.002)** | -0.169 (0.057) | -0.178 **(0.044)** | 0.272 **(0.002)** | -0.100 (0.262) |
| **Hallucinations** | -0.249 **(0.006)** | 0.121 (0.189) | 0.162 (0.078) | -0.222 **(0.017)** | 0.157 (0.093) | -0.274 **(0.002)** | -0.013 (0.888) | 0.160 (0.070) | 0.011 (0.901) |
| **Headaches** | -0.123 (0.182) | 0.274 **(0.003)** | 0.269 **(0.003)** | -0.186 **(0.045)** | 0.315 **(0.001)** | -0.077 (0.392) | -0.166 (0.061) | 0.222 **(0.011)** | -0.152 (0.088) |
| **Insomnia** | -0.104 (0.259) | 0.213 **(0.020)** | 0.146 (0.113) | -0.177 (0.057) | 0.253 **(0.006)** | -0.075 (0.400) | -0.004 (0.966) | 0.151 (0.087) | -0.056 (0.532) |
| **Irritability** | -0.099 (0.286) | 0.141 (0.127) | 0.168 (0.068) | -0.188 **(0.043)** | 0.240 **(0.009)** | -0.109 (0.223) | -0.097 (0.276) | 0.129 (0.146) | -0.029 (0.749) |
| **Lack of appetite** | -0.047 (0.611) | 0.185 **(0.044)** | 0.108 (0.241) | -0.132 (0.158) | 0.189 **(0.043)** | -0.129 (0.148) | -0.066 (0.459) | 0.253 **(0.004)** | -0.143 (0.109) |
| **Loss of taste and/or smell** | -0.059 (0.523) | 0.295 **(0.001)** | 0.091 (0.326) | -0.137 (0.143) | 0.295 **(0.001)** | -0.145 (0.105) | -0.013 (0.888) | 0.189 **(0.032)** | -0.083 (0.351) |
| **Mild cognitive problems** | -0.050 (0.586) | 0.212 **(0.021)** | 0.131 (0.157) | -0.176 (0.059) | 0.360 **(<0.001)** | -0.164 (0.065) | -0.132 (0.135) | 0.293 **(0.001)** | -0.099 (0.266) |
| **Muscle/body ache** | -0.062 (0.506) | 0.374 **(<0.001)** | 0.180 **(0.050)** | -0.180 (0.053) | 0.373 **(<0.001)** | -0.219 **(0.013)** | -0.170 (0.054) | 0.345 **(<0.001)** | -0.116 (0.195) |
| **Sore eyes/conjunctivitis** | -0.086 (0.354) | 0.303 **(0.001)** | 0.116 (0.208) | -0.112 (0.229) | 0.276 **(0.003)** | -0.168 (0.059) | -0.046 (0.602) | 0.261 **(0.003)** | 0.007 (0.940) |
| **Sore throat** | -0.014 (0.877) | 0.075 (0.418) | 0.011 (0.907) | -0.039 (0.677) | 0.055 (0.555) | -0.034 (0.702) | -0.083 (0.353) | 0.187 **(0.034)** | -0.054 (0.546) |
| **Temperature** | -0.118 (0.203) | -0.003 (0.973) | 0.080 (0.386) | -0.135 (0.149) | 0.063 (0.500) | -0.111 (0.214) | -0.121 (0.171) | 0.176 **(0.046)** | -0.005 (0.854) |
| **Vomiting/nausea** | -0.096 (0.299) | 0.232 **(0.011)** | 0.144 (0.119) | -0.271 **(0.003)** | 0.157 (0.093) | -0.104 (0.246) | -0.189 **(0.032)** | 0.274 **(0.002)** | -0.052 (0.561) |
